# Epidural analgesia in cattle, buffalo, and camels

**DOI:** 10.14202/vetworld.2016.1450-1455

**Published:** 2016-12-19

**Authors:** Zuhair Bani Ismail

**Affiliations:** Department of Veterinary Clinical Sciences, Faculty of Veterinary Medicine, Jordan University of Science and Technology, P. O. Box: 3030, Irbid 22110, Jordan

**Keywords:** buffalo, camel, cattle, epidural analgesia, side effects

## Abstract

Epidural analgesia is commonly used in large animals. It is an easy, cheap, and effective technique used to prevent or control pain during surgeries involving the tail, anus, vulva, perineum, caudal udder, scrotum, and upper hind limbs. The objectives of this article were to comprehensively review and summarize all scientific data available in the literature on new techniques and drugs or drug combinations used for epidural anesthesia in cattle, camel, and buffalo. Only articles published between 2006 and 2016 were included in the review. The most common sites for epidural administration in cattle, camels, and buffalos were the sacrococcygeal intervertebral space (S5-Co1) and first intercoccygeal intervertebral space (Co1-Co2). The most frequently used drugs and dosages were lidocaine (0.22-0.5 mg/kg), bupivacaine (0.125 mg/kg), ropivacaine (0.11 mg/kg), xylazine (0.05 mg/kg), medetomidine (15 µg/kg), romifidine (30-50 µg/kg), ketamine (0.3-2.5 mg/kg), tramadol (1 mg/kg), and neostigmine (10 µg/kg), and the clinical applications, clinical effects, recommendations, and side effects were discussed.

## Introduction

Ruminants are poor candidates for general anesthesia because of the increased risk of complications such as regurgitation, bloating, and muscle damage [1]. Therefore, surgical interventions under local anesthesia in standing animals are preferred [1]. Paravertebral nerve block, local infiltration of anesthetic agents, intravenous regional limb perfusion, and epidural anesthesia are commonly used in ruminant surgery [1]. Various obstetrical operations, surgical procedures of the anus, vulva, perineum, caudal udder, and scrotum are performed under epidural analgesia. Epidural analgesia is also used as an adjunct for the treatment and control of tenesmus [1,2].

Caudal epidural analgesia has received plenty of research over the last 10 years. Many anesthetic drugs and combinations thereof have been experimented in ruminants with variable successful results. By searching the current literature, however, no comprehensive review articles that summarize published findings regarding epidural analgesia in cattle, camels, and buffalos can be found. Therefore, the objectives of this article were to comprehensively review and summarize all scientific data available in the literature on techniques and drugs or drug combinations used for epidural anesthesia in cattle, camel, and buffalo. Databases - such as Google scholar, Pubmed, and ResearchGate - were used to find newly published articles. Keywords such as ruminant anesthesia, local anesthesia in ruminants, epidural analgesia in cattle, buffalo, and camels were used to find articles in those databases. Only articles published between 2006 and 2016 were included in the review. Only research article using an epidural injection of drugs (extradural) was included in the study. Data regarding the technique of epidural administration, drugs and drug combinations, dosages, age categories (young vs. adult), species and breed, side effects and recommendations for applications of various techniques were discussed.

## Epidural Injection Techniques and Classification

In large animals, the most common sites for epidural administration of anesthetic agents are the first coccygeal intervertebral space (Co1-Co2) and the sacrococcygeal intervertebral space (S5-Co1) [3,4]. The technique is considered easy to perform in standing animals and require no special equipment. The site of injection can be identified by moving the tail up and down in a pump-like manner. The first proximal moving space that can be easily palpated is the preferred location for injection [3]. The site in the dorsal midline is clipped and aseptically prepared using a disinfectant solution. An 18-Gauge, 1.25″ needle is used to penetrate the intervertebral space [3]. The needle is usually directed slightly in a cranial direction and advanced slowly. A lack of resistance or popping sensation usually indicates that the epidural space is entered [3]. Correct placement of the needle can be checked by the hanging drop technique which can be performed by placing few drops of sterile water or lidocaine into the needle hub during insertion [3]. When the needle enters the correct space, the drop of saline or lidocaine is observed to be aspirated under the effect of the negative pressure in the epidural space [3]. Furthermore before injection of the drug, negative pressure is applied by the syringe to ensure blood or spinal fluid is not aspirated [3]. In which case, the needle must be withdrawn and adjusted slightly and negative pressure is applied again [3].

According to the volume of injected drug, epidural anesthesia can be classified into caudal (low dose or low volume) epidural or cranial (high dose or high volume) epidural [3]. Low dose or caudal epidural anesthesia is the most commonly used technique and it requires the injection of a small volume of the drug [3]. This technique desensitizes the caudal sacral nerves within the spinal canal. The motor functions of the hind limbs are not affected [3]. Areas that are desensitized by low volume epidural are the tail, vagina, vulva, anus, rectum, caudal prepuce, scrotum, and urethra [3]. This technique is commonly used to prevent or control tenesmus and contractions during repair of a prolapsed rectum or vulva, repositioning of a prolapsed uterus, and dystocia [3].

In the high dose epidural anesthesia technique, the volume of the injected drug is relatively large and analgesia is extended therefore further cranially [1,3]. Analgesia may reach up to the diaphragm resulting in some degree of cardiopulmonary compromise [1,3]. In addition, the motor functions of the hind limbs will be affected resulting in ataxia and recumbency in some animals [1,3]. This technique is less frequently used in adult animals, however in young calves, it is may be used for umbilical surgeries [1,3].

## Drugs and Drug Combinations

The most frequently used drugs for epidural analgesia in cattle, camel, and buffalo (Table-1) were lidocaine, bupivacaine, ropivacaine, xylazine, medetomidine, romifidine, ketamine, tramadol, and neostigmine.

**Table-1:** Anesthetic drugs, dosages, analgesic effects and side effects used in cattle, camels, and buffalos for epidural analgesia.

Drugs	Dosages	Technique	Species	Findings/side effects	References
Bupivacaine (mg/kg)	0.125	Co1-Co2	Buffalo calves	Mild to moderate analgesia Mild to moderate ataxia	[15]
Ropivacaine (mg/kg)	0.11	Co1-Co2	Adult cows	Variable analgesia with minimal ataxia	[16]
Lidocaine (mg/kg)	0.22	S5-Co1Co1-Co2	Buffalo calves; adult buffalo; camel calves; cattle calves	Adequate analgesia of the mild to moderate ataxia	[6,9,11,23]
Lidocaine (mg/kg)	0.5	Co1-Co2	Cattle calves	Mild to moderate analgesia	[13]
Neostigmine (μg/kg)	10	S5-Co1	Buffalo calves	Adequate analgesia of the mild or moderate ataxia	[23]
Xylazine (mg/kg)	0.05	S5-Co1 Co1-Co2	Buffalo	Analgesia ascended to thoracic segments Mild to moderate ataxia	[6,15,17]
Tramadol (mg/kg)	1.0	Co1-Co2	Adult Holstein cows	Combine with lidocaine is recommended	[8]
		S5-Co1	Buffalo calves	Adequate analgesia in combination with lidocaine	[11]
Medetomidine (μg/kg)	15	Co1-Co2	Buffalo calves	Adequate analgesia of the mild ataxia	[15]
Ketamine (mg/kg)	0.3, 0.5, 0.7, 2.5	L6-S1 Co1-Co2	Buffalo calves Adult cattle	Complete analgesia of the flank region Mild to moderate ataxia	[17,21]
Romifidine (μg/kg)	30, 40, 50	Co1-Co2	Adult cattle	Dosedependent analgesia and sedation	[20]

The most common drug combinations used for epidural analgesia in cattle, camel, and buffalo (Table-2) were lidocaine-xylazine, lidocaine-tramadol, lidocaine-ketamine, lidocaine-magnesium sulfate (MgSO_4_), xylazine-ketamine, ketamine-medetomidine, xylazine-bupivacaine, and medetomidine-bupivacaine.

**Table-2:** Combinations, dosages, analgesic effects and side effects of anesthetic drugs used in cattle, camels, and buffalos for epidural analgesia.

Drugs	Dosages	Technique	Species	Findings/side effects	References
Lidocaine-Xylazine (mg/kg)	0.22 and 0.05	S5-Co1	Adult buffalo	Adequate analgesia Mild to moderate ataxia	[6]
Lidocaine-tramadol (mg/kg)	0.22 and 1	Co1-Co2	Camel calves	Adequate analgesia Mild to moderate sedation and severe ataxia	[10]
Lidocaine-tramadol (mg/kg)	0.11 and 0.5	Co1-Co2 S5-Co1	Adult Holstein cows Buffalo calves	Adequate analgesia Mild ataxia in buffalo	[8,11]
Lidocaine-ketamine (mg/kg)	0.22 and 1	Co1-Co2	Camel calves	Adequate analgesia Mild ataxia	[9]
Lidocaine (mg/kg)-MgSO_4_(10%)	0.22 and 1 ml	S5-Co1	Adult Holstein cows	Adequate analgesia	[7,8,13]
Lidocaine-ketamine (mg/kg)	0.5 and 2	Co1-Co2	Young calves	Adequate analgesia Moderate ataxia	[13]
Xylazine-ketamine (mg/kg)	0.05 and 2.5	L6-S1	Buffalo calves	Adequate analgesia Severe ataxia	[17]
Ketamine-xylazine (mg/kg)	2.5 and 0.17	Co1-Co2	Adult camels	Adequate analgesia Mild ataxia and moderate sedation	[18]
Ketamine (mg/kg)-medetomidine (μg/kg)	2.5 and 10		Adult camels	Long duration of analgesia Mild ataxia and moderate sedation	[18]
Xylazine-bupivacaine (mg/kg)	0.05 and 0.125	Co1-Co2	Buffalo Calves	Adequate analgesia Mild to moderate ataxia	[15]
Medetomidine (μg/kg)-bupivacaine (mg/kg)	15 and 0.125	Co1-Co2	Buffalo calves	Adequate analgesia Mild to moderate ataxia sedation	[15]

MgSO_4_= Magnesium sulfate

## Lidocaine

Lidocaine is an amino amide-type of local anesthetic. It acts by blocking signal conduction by altering the fast voltage-gated sodium channels at the neuronal cell membrane [5]. However, because its action is not specific to the sensory tracts, it also blocks motor and sympathetic fibers causing hind limb ataxia and weakness and sometimes recumbency [1,3]. The duration of action is short which is not suitable in long-duration surgical operations making readministration necessary to complete long surgical procedures [1,3]. Opioids and alpha-2 adrenergic agonists are commonly used in combinations with lidocaine resulting in longer and adequate analgesia [1,3,6-11].

Lidocaine hydrochloride (2%) was the most commonly used local anesthetic drug for epidural analgesia in cattle, camel, and buffalo (Table-1). The dose ranged between 0.11 and 0.22 mg/kg of 2% solution [6,9,11]. Most adult cows required between 5 and 6 ml of the drug to achieve adequate surgical analgesia of the caudal regions of the body. Larger volumes were associated with increased risk of motor paralysis to the hind limbs leading to ataxia, incoordination, and recumbency. In most of the studies, occurrence if analgesia with lidocaine was within 3-5 minutes after injection and lasting up to 150 minutes depending on the dose. For high dose epidurals, the maximum dose of lidocaine was up to 2-8 mg/kg body weight [12].

Alone or more commonly in combination with other anesthetic agents such as ketamine, lidocaine produced adequate surgical analgesia of the tail base, anus, and perineum in calves [13]. However, ataxia of the hind limbs was more pronounced when lidocaine was used in combination with ketamine [13].

## Bupivacaine

Bupivacaine is a potent amino-amine local anesthetic with an extended duration, lower degree of motor blockade and lesser toxic effects [14]. In buffalos, bupivacaine produced complete analgesia of the tail, perineum, inguinal, and thigh regions. However, when used in combination with either xylazine or medetomidine, bupivacaine resulted in enhanced analgesia produced by xylazine, but not medetomidine [15].

## Ropivacaine

Ropivacaine, a long-acting amino-amine local anesthetic agent was used (0.75%, 0.11 mg/kg) for epidural analgesia in adult cows [16]. It resulted in a prolonged bilateral perineal analgesia with minimal ataxia and cardiopulmonary effects in standing cattle [16].

## Alpha-2 Agonists

Xylazine and medetomidine are common alpha-2 agonist sedative and analgesic drugs. Alpha-2 agonists produce their analgesic effects by stimulating the alpha-2 adrenergic receptors in the dorsal horn of the spinal cord [15]. Xylazine alone or in combination with lidocaine is the second most commonly used drug for epidural anesthesia in cattle [15-18]. It resulted in longer duration of anesthesia using the low dose epidural technique. Alone, xylazine is used at a dose rate of 0.05 mg/kg (2%) diluted into 5 ml sterile water [15-18]. Onset and duration of anesthesia is usually within 10 min and 3-4 h, respectively. Longer duration of anesthesia, however, is expected when xylazine (0.03-0.05 mg/kg) is combined with lidocaine [6,18]. The onset of anesthesia and duration of this combination is 5 min and 6 h, respectively [6,18]. Epidural xylazine also results in mild to moderate sedation and mild ataxia [6,18]. There is also an increased risk of recumbency, decreased ruminal motility and bradycardia [6,18].

In one study in bulls undergoing semen collection using an electroejaculator, xylazine plus hyaluronidase was shown to reduce discomfort during the procedure more effectively than xylazine or lidocaine alone [19].

Epidural administration of medetomidine can produce complete analgesia of the tail, perineum, inguinal region, and upper hind limbs in buffaloes. However, significant depression of cardiovascular parameters was recorded [15].

Administration of ketamine along with medetomidine resulted in significantly early onset and slightly longer duration of analgesia with lesser cardiopulmonary side-effects compared to medetomidine alone or medetomidine with bupivacaine [15]. Although the addition of ketamine to medetomidine enhanced epidural analgesia, the addition of bupivacaine failed to provide any advantage over medetomidine alone [15].

In adult camels, epidural administration of ketamine and xylazine combination was compared to that induced by ketamine and medetomidine combination [18]. Analgesia was slower in onset and longer in duration after ketamine and medetomidine combination. Mild ataxia and moderate degree of sedation were reported with ketamine-xylazine and ketamine-medetomidine combinations for variable length of times. It was concluded that the combination of ketamine with medetomidine results in a superior analgesic effect than that produced by ketamine-xylazine combination in camels.

Romifidine is an imidazolidine derivative and an alpha-2 agonist [20]. It has been used in various animal species including horses, dogs, goats, and cattle for its systemic and analgesic effects [20]. In cattle, when administered systemically, romifidine appears to have similar effects to xylazine with faster and longer duration of analgesia [20]. After epidural administration of romifidine in adult cattle, the antinociceptive effect was produced in the tail, anus, perineum, vulva, and inguinal area [20]. Analgesia was reported to extend to the coronary band of the hind limbs and cranially to the thoracic areas [20]. A dose-dependent sedative effect was reported with mild to moderate sedation achieved at 30 and 40 μg/kg doses and deep sedation at the 50 µg/kg dose following epidural administration [20].

## Tramadol

Tramadol is a synthetic analog of codeine [10]. The mechanism of action of tramadol is attributed to its interaction with opioid μ receptors in the brain and spinal cord. It also modulates the monoaminergic spinal pain by inhibiting the re-uptake of norepinephrine and serotonin [10].

Tramadol has not been tested alone in any of the animal species as an analgesic agent, however, lidocaine in combination with tramadol (LT) has been used in camel calves and in adult Holstein cows and in buffalo calves [8,10,11]. LT in camel and buffalo calves produced complete analgesia in the tail, anus, and perineum in addition to a mild to moderate sedation [10,11]. However, animals that received LT experienced much more ataxia than those that received lidocaine alone. Similar results, but without the ataxia, were also obtained in adult Holstein dairy cows that received LT combination [8]. It was concluded that in cattle, LT combination could be used for long-duration obstetrical and surgical procedures of the perineal area without the need for re-administration of anesthetic agents [10].

Analgesia induced by tramadol administration was dose dependent [10]. Slight to mild sedation and ataxia were observed when cows received 2 or 3 mg of tramadol/kg. No significant tramadol-associated changes in heart rate, respiratory rate, rectal temperature, or rumen motility were detected [10]. In another study, the duration of analgesia produced by tramadol-lidocaine combination was significantly longer than lidocaine alone. Ataxia was mildly observed in LT and was moderate in lidocaine [11].

## Ketamine

Ketamine is a noncompetitive antagonist of N-methyl-D-aspartate (NMDA) receptors in the spinal cord [9]. Ketamine induces local analgesia by blocking the sodium ion channels. Centrally, it also interacts with opioid, monoaminergic and muscarinic receptors and voltage-sensitive calcium ion channels [9].

Epidural administration of ketamine alone or in combination with lidocaine or other sedative agents has been reported to produce perineal analgesia in cattle, water buffaloes, and camels [9,13,17,18,21].

In camel calves, lidocaine with ketamine (LK) produced adequate analgesia of the perineal area and moderate sedation [9]. Although ataxia was observed in all subjects, it was more pronounced in LK calves. The analgesia was of significantly longer duration in standing camels compared with the effect of lidocaine alone.

## MgSO_4_

Magnesium is endogenous cation in the body that plays many physiological actions. In addition, when administered centrally, it acts as an antinociceptive in both human and animal models of pain [22]. The analgesic effects of magnesium are believed to be due to the regulation of calcium influx into the cell which acts as a calcium antagonist and also due to its antagonistic effect on NMDA receptors [22]. MgSO_4_ has been used for several years as an adjunct to post-operative pain medication in human surgery [22].

In large animals, data regarding the use of magnesium for epidural anesthesia is almost not available. In one study, caudal epidural anesthesia was obtained in adult dairy cows using a combination of 2% lidocaine (0.22 mg/kg) solution and 1 mL of 10% MgSO_4_ solution [7]. Epidural administration of lidocaine-MgSO_4_ produced adequate analgesia of significantly longer duration of the perineal area than that of lidocaine-distilled water [7].

## Neostigmine

Neostigmine is a cholinesterase inhibitor that inhibits the breakdown of endogenous acetylcholine and thus indirectly stimulates both muscarinic and nicotinic receptors [23]. Although it has been used in human surgery as an adjunct to post-operative pain medications, there are only a few studies that described its use for epidural analgesia in large animals [24]. It has been shown that neostigmine prolongs the duration of epidural analgesia in dogs and cattle compared with lidocaine alone [8,25]. In horses, neostigmine along with lidocaine improved and extended the duration of analgesia in the perineal region [26].

## Conclusion

This review article collected relevant information regarding most recent drugs, drug combinations, and techniques used in clinical trials for epidural injections in cattle, camel, and buffalo. Clinical applications, drug dosages, and side effects were discussed.

## Competing Interests

The author declare that they have no competing interests.
